# Study protocol: Randomised controlled trial to evaluate the impact of an educational programme on Alzheimer’s disease patients’ quality of life

**DOI:** 10.1186/s13195-014-0066-1

**Published:** 2014-10-27

**Authors:** Hélène Villars, Virginie Gardette, Amélie Perrin, Christophe Hein, Sophie Elmalem, Eva de Peretti, Audrey Zueras, Bruno Vellas, Fati Nourhashémi

**Affiliations:** 1 Geriatric Department, University Hospital, 170 avenue de Casselardit, Toulouse Cedex, 31059, France; 2 Department of Epidemiology and Public Health, Adresse 37, allées Jules Guesde, Toulouse Cedex, 31073, France; 3 Inserm U 1027, University Toulouse III, Toulouse, F-31073, France

## Abstract

**Introduction:**

Therapeutic education is expanding in the management of Alzheimer’s disease (AD) patients. Several studies have revealed a positive impact of therapeutic educational programmes on the caregiver’s burden and/or quality of life. However, to date, no study has evaluated its impact on the quality of life of the AD patient.

**Methods:**

The THERAD study (THerapeutic Education in Alzheimer’s Disease) is a 12-month randomised controlled trial that started in January 2013. This paper describes the study protocol. THERAD plans to enroll 170 dyads (AD patient and caregiver) on the basis of the following criteria: patient at a mild to moderately severe stage of AD, living at home, receiving support from a family caregiver. The main outcome is the patient’s quality of life assessed by the Logsdon QoL-AD scale at 2 months, reported by the caregiver. The study is being led by geriatricians trained in therapeutic education at Toulouse University Hospital in France. To date, 107 caregiver/patient dyads have been recruited.

**Conclusion:**

This is the first trial designed to assess the specific impact of a therapeutic educational programme on the AD patient’s quality of life. The final results will be available in 2015.

**Trial registration:**

[ClinicalTrials.gov: NCT01796314] Registered 19 February 2013

## Introduction

Therapeutic education in the care of Alzheimer’s disease (AD)-affected patients has only recently been instigated [1]-[8]. Indeed, it seems difficult to implement such programmes because of the following two characteristics of the disease: memory loss and anosognosia. The patient’s abilities to acquire skills and modify his/her behaviour are often deeply affected by the disease. The caregiver, who bears a substantial burden that can have adverse effects on his/her physical and mental health, consequently appears to be the real beneficiary of those approaches [5],[6]. For this reason, most of the studies in the field of therapeutic education in AD have focused on the impact of therapeutic educational programmes on the caregiver’s outcomes [2],[4]-[8]. Several works have demonstrated positive results, such as an improvement in the caregiver’s quality of life (QoL) [7] and a decrease in their depression [5],[6] and burden [2]. As regards the AD patient, a few studies have evaluated the effectiveness of psychoeducational interventions on his/her behaviour [9], functional autonomy [3] or cognitive status [1]. The large majority of studies have evaluated multidimensional interventions including educational activities (associated, as appropriate, with respite, psychological support and/or pharmacological treatment). Two trials showed that this type of multidimensional intervention decreases the behavioural and psychological symptoms of dementia [9],[10].

A meta-analysis suggests that the most effective interventions for both patient (behavioural and psychological symptoms of dementia) and caregiver (burden) are those with an intensive psychoeducational programme for caregivers accompanied by a follow-up [11]. In a literature review [12], four studies demonstrated positive results of combined intervention programmes including psychoeducational components on both patients and caregivers [13]-[16]. In these programmes, the mental health of the AD patient was improved [13],[14] and admission to long-stay care was delayed [15],[16]. Lastly, among more recent studies, the Danish Alzheimer Intervention Study randomised controlled trial conducted in Denmark has demonstrated that a psychosocial counselling and support programme for outpatients with mild AD and their primary caregivers leads to a small, although nonsignificant, difference in the patient’s depression (assessed by the Cornell depression scale score [17]) in the intervention group [1].

These studies have not specifically studied the patients’ QoL as an outcome – except for the Danish Alzheimer Intervention Study, but its intervention was not exclusively educative – and were not methodologically designed for this purpose. In the Therapeutic Education in Alzheimer’s Disease (THERAD) study we chose to evaluate the impact of an educational programme on the AD patients’ QoL, given the results in the literature and those of a feasibility study we have conducted. QoL seems to be a global and relevant criterion that meets the overall objectives of any therapeutic educational action in the care of dementia as well as in most chronic diseases [18],[19]. However, the validity of self-reported QoL assessments for demented patients is a critical issue [20]. Many questions have been raised about the ability of patients with dementia to estimate their QoL [20]. For this reason, self-rating of the QoL is replaced by proxy rating in a large majority of clinical trials involving AD patients, and can be considered a valid outcome [19],[21],[22]. In the THERAD study, we also chose to use the caregiver’s rating of the QoL. We will also raise the reasons for our choice in the Discussion. It has been shown that a multidimensional approach including, but not involving only, therapeutic education could improve the AD patient’s QoL [23].

For all these reasons, it was reasonable to assume that a better understanding of the disease by the family caregiver can have a positive impact on his/her coping strategies and indirectly on the AD patient’s QoL. We therefore designed the THERAD study. The trial addresses community-dwelling patients suffering from mild to moderately severe AD who receive support from a family caregiver. This paper describes the study protocol. In our opinion, when attempting to support the patient/caregiver dyad in AD, therapeutic education is expected to be an especially effective tool, as well as in many chronic diseases [24],[25].

## Methods

### Study design

**Figure 1:**
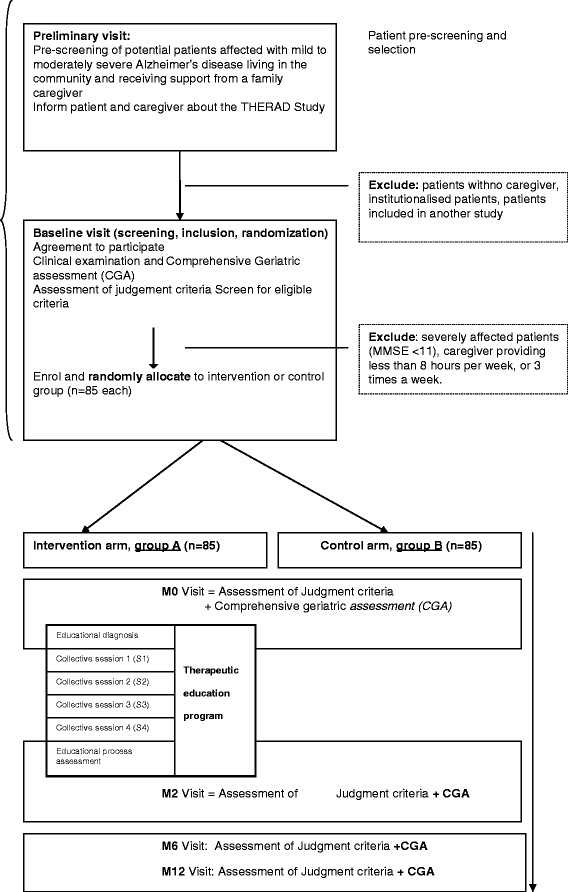
**Entry and follow-up procedures of the THERAD study.** MMSE, Mini-Mental State Examination; THERAD, Therapeutic Education in Alzheimer’s Disease.

The THERAD study is a monocentric, randomised, single-blind, controlled trial and has been registered on clinicaltrials.gov since 19 February 2013 [ClinicalTrials.gov: NCT01796314]. The AD patient/caregiver dyads in the intervention group receive a therapeutic educational programme and the dyads in the control group benefit from usual care. The THERAD study will enrol 170 dyads, 85 in the intervention group and 85 in the control group. The entry and follow-up procedures are illustrated in Figure 1. The study protocol was approved by the French Ministry of Health. Informed consent was obtained from all enrolled participants. The Toulouse University Hospital has delivered ethical approval. Blinding of raters is performed in a single-blind experimental design: participants know the full facts (group session), but not the experimenters. The randomisation was done with STATA® Version 11.0. We performed a blocked randomisation. We used a block of four, of fixed size, without stratification. The allocation procedure was conducted by the Department of Epidemiology of the Toulouse University Hospital in Toulouse, France. The randomisation list is printed and kept in this department, protected by a password. At enrolment, a fax is sent by the THERAD study group to this department, together with the participant code and information regarding assignment. The investigators are unaware of the randomisation list. The assignment is concealed for the raters at follow-up visits.

### Participants

AD patients suffering from mild to moderately severe stages of the disease and their primary caregivers are eligible for inclusion. The diagnosis of AD is based on the clinical criteria of AD according to the *Diagnostic and Statistical Manual of Mental Disorders* (fourth edition) [26]. Each patient undergoes magnetic resonance imaging or a computed tomography scan and biological tests. We are targeting mild to moderately severe stages of the disease defined by the Mini-Mental Score Examination score [27], with scores of 26 to 11/30. We are enrolling community-dwelling patients receiving support from a family caregiver. The caregiver is defined as a nonprofessional person living with the patient or providing support to him/her at least three times a week or for 8 hours a week. Patients of both sexes with no age limit are eligible. Participants are being recruited from the memory clinic and the geriatric units of the Toulouse University Hospital in France. The inclusion period began in January 2013, with an expected duration of 18 months. To date, 107 dyads have been enrolled.

### Sample size

We hypothesise a similar QoL score in the two groups at baseline, 25 ± 5.5 points (Logsdon Quality of Life in Alzheimer’s disease (QoL-AD) scale including 52 points [28]). To detect an increase of 5% in the total score and a relative variation of more than 10% of the baseline score between trial arms, with an alpha risk of 5% and a beta risk of 20%, and anticipating 20% attrition, 85 dyads are required per group. The total sample size required for the study is 170 dyads (85 per arm).

### Primary outcome measure

Our primary outcome measure is a change in the patient’s QoL at 2 months, determined by the Logsdon QoL-AD scale, assessed by the primary caregiver [28]. We chose the patient’s QoL criterion because it is a multidimensional criterion that can be assessed by validated tool. We chose the Logsdon QoL-AD scale because it has been validated in our population [29]. This scale can be completed by the patient or the caregiver.

Considering that self-report could be difficult for mild to moderately severe AD patients, we chose to use hetero-evaluation by the caregiver as primary outcome, as in many clinical trials involving AD dyads and targeting the patient’s well-being [30]. The caregivers are instructed to carry out the rating of the QoL according to the instructions for interviewers provided by Logsdon and colleagues [28]. They are asked to fill in the scale as a self-administered 13-item questionnaire, concerning their relative’s QoL. We tried to minimise the risk of an experimenter’s bias by using a method of collection of data that does not require active involvement of an interviewer.

Nevertheless, we are collecting the self-assessed Logsdon QoL-AD as a secondary outcome.

### Secondary outcome measure

The secondary endpoints are: the frequency and severity of the patient’s behavioural and psychological symptoms of dementia assessed by the NeuroPsychiatric Inventory [31]; the patient’s functional autonomy assessed by the Activities of Daily Living scale [32] and the Instrumental Activities of Daily Living scale [33]; the patient’s QoL assessed by the Logsdon QoL-AD scale, self-reported; the primary caregiver’s burden assessed by the Zarit Burden Inventory [34]; the primary caregiver’s QoL assessed by the Nottingham Health Profile [35]; and the primary caregiver’s mood assessed by the mini-Geriatric Depression Scale [36].

We are collecting these data at 2 months, 6 months and 12 months. Lastly, we are collecting information on factors that can influence QoL, as demonstrated in the literature, such as acute disease for both the patient and his/her primary caregiver, change in home care support, hospitalisation or institutionalisation [37].

### Detailed study scheme

The entry and follow-up procedures are illustrated in Figure 1.

All visits are being performed by geriatricians who are hospital practitioners trained in therapeutic education. Written agreement to participate is being obtained from all participants (patients and caregivers).

The baseline visit (M0) concerns both the patient and the caregiver. In both the intervention group (group A) and the control group (group B), the patient receives a consultation led by a geriatrician. This includes a clinical examination and comprehensive geriatric assessment (Mini-Mental Score Examination, weight, one-leg balance, sensorial impairment, co-morbidities, pharmacological and nonpharmacological therapies) [38]. The outcomes concerning the patient are assessed at baseline by: Logsdon QoL-AD reported by the primary caregiver and patient; and the Activities of Daily Living, the Instrumental Activities of Daily Living and the NeuroPsychiatric Inventory reported by the caregiver. The secondary outcomes concerning the caregiver are assessed by the mini-Geriatric Depression Scale, the Zarit Burden Interview and the Nottingham Health Profile, self-reported.

At the end of the baseline visit, each patient/caregiver dyad is randomly allocated to group A or group B (Figure 1). The random number is generated by a computerised generator using block randomisation. The block size is concealed from all researchers. As described in the intervention section and in Figure 1, at the end of the baseline visit the dyads of the intervention group (group A) receive the first step of the intervention: the educational diagnosis (cf. Intervention).

The second 2-month visit (M2) also involves the dyads of both groups A and B, but the first part differs between groups. As described in Intervention, at the beginning of M2 the participants in the intervention group (group A) benefit from the last part of the intervention and its assessment. The participants of both groups then receive a clinical examination, comprehensive geriatric assessment and assessment of endpoints.

Participants in groups A and B receive a follow-up visit at 6 months and at 12 months, including the assessment of the primary and secondary endpoints.

### Intervention

The intervention consists of a therapeutic educational programme. The total duration of intervention is 2 months. The intervention is composed of two individual sessions (M0 and M2) (for both patients and caregivers) and four group sessions (for caregivers only) between M0 and M2.

The first individual session – the educational diagnosis – takes place at the end of M0. Indeed, the educational diagnosis is the first step of any educational process [39],[40]. Via this specific stage, the educational team can better understand the different aspects of the patient’s personality and life history, and those of the caregiver as well. This stage allows the team to identify their needs, assess their potential and consider their requests. Its major purpose is to help the dyad to formulate a project by identifying skills to acquire or strengthen and defining realistic goals to reach [40]. The patient participates in this individual session because it is important to collect their representations and beliefs about the disease, which are useful for the educational process of the dyad. As a matter of fact, many people with dementia do not have the opportunity to raise their concerns once a diagnosis is made, and often feel isolated [41]. The AD patient’s educational diagnosis is supported by a specific tool, developed by the French Ministry of Health [42]. This tool is a booklet specifically designed for patients. It is performed as a semi-directive interview with patient-centred communication techniques, and educational techniques. The patient can also note his/her thoughts about daily life in the booklet. This educational diagnosis is then linked with that of the caregiver so that the team can establish a semi-tailored plan with learning priorities for the caregiver depending on the patient’s educational diagnosis (for example, to adapt his/her communication style in stressful situations). The intervention is supported by the use of a monitoring scheme established at this step, used during the follow-up sessions. Indeed, therapeutic patient education is a continuing process adjusted to disease course and patient lifestyle. A formal document is delivered to patients, called the ‘Alzheimer’s Card’, also developed by the French Ministry of Health [43]. This card is kept by the patient. It includes information on disease management (for example, name of the family practitioner, drugs taken, telephone number of the relatives) and provides counselling in legible and understandable form for cognitively impaired subjects.

Then caregivers benefit from four group sessions, one per week for a month. These sessions are conducted in small groups (six to eight caregivers) and last 3 hours each. They aim towards allowing people to gain better understanding of their relative’s illness, have their concerns addressed and have their feelings expressed. We have planned four group sessions for caregivers, each focusing on a specific aspect of the disease: Session 1 (S1) – knowledge of the disease, understanding of the symptoms, functional abilities; Session 2 (S2) – treatment (pharmacological and nonpharmacological aspects), behavioural and psychological symptoms of dementia in daily living; Session 3 (S3) – management of crisis situations and prevention of caregiver’s exhaustion; and Session 4 (S4) – assistive devices, home care support and care pathways. Table 1 describes the objectives and topics of the group sessions.

**Table 1:** Educational objectives of the group sessions in the intervention group of the THERAD study

	**Objectives**
Session 1 (S1): knowledge of the disease	- To identify the cognitive impairment of the patient in everyday life
	- To recognise the remaining functional abilities
	- To determine the nonpharmacological measures to be implemented
Session 2 (S2): pharmacological and nonpharmacological treatments. Behavioural and psychological symptoms of dementia	- To understand the potential effect of pharmacological therapies
	- To identify behavioural and psychological symptoms of the disease
	- To implement nonpharmacological measures to prevent these symptoms
Session 3 (S3): crisis situations. Prevention of caregiver’s exhaustion	- To identify conditions of high risk (treatment modification, concomitant illness, change of environment, relocation)
	- To recognise and prevent caregiver’s burnout
	- To identify their resources
Session 4 (S4): assistive devices and care pathways	- To understand assistive devices, respite and legal aspects raised by the disease
	- To identify the resources available in crisis or emergency situations (general practitioner, specialist consultation, hospitalisation)

THERAD, Therapeutic Education in Alzheimer’s Disease.

Each session is adaptable to the objectives of the participants in each group. The goal we intend to achieve during these group sessions is to develop the caregivers’ coping strategies. It is also part of the educational process to offer them emotional support.

The sessions are led by a geriatrician (S1 and S2) and a nurse (S2, S3 and S4), both trained in therapeutic education, in tandem with stakeholders belonging to the geriatric department: a pharmacist (S2), a psychologist (S3) and a social worker (S4). The pedagogic methods and tools are those currently used in therapeutic educational programmes [40]. We use tools such as storytelling or drawings that allow us to explore the subject’s representations and worries [44]. We also use computer-based activities and brainstorming, as commonly used in health education.

Patients can also receive written documents, when needed, to support the educative process delivered to their relatives during group sessions.

After the group session, the dyads benefit from an individual visit at M2. This is the continuation of the educational process with a reformulation of their personal objectives: ‘achieved’, ‘to reach’, ‘new goals’ and ‘acquired skills’. We try to deliver additional advice based on this evaluation, to both the patient and the caregiver. The patient is interviewed, using the booklet, on his/her well-being.

We are also collecting data regarding the participants’ satisfaction. These qualitative data are part of the educational process.

### Analysis

Data are collected in the Access database and SAS software (developped by the SAS institute based in Cary, North Carolina, USA) will be used to perform the statistical analysis. Intention-to-treat analysis using a linear mixed model will be performed, adjusting for patient (disease severity) and caregiver (burden) characteristics and occurrence of life events that may interfere with the patient’s QoL. Attrition of dyads has been taken into account. At the moment, at the 12-month visit, there is less than 5% attrition. Moreover, the total sample size required for the study was calculated anticipating a risk of 20% attrition to take into account this phenomenon, which is particularly important in our population [45].

## Discussion

The THERAD study started in January 2013. To date, 107 dyads have been included. The final results will be available in 2015. By transmitting knowledge, expertise and skills to the AD patient’s caregiver [46], we hypothesised that the patient’s QoL can improve. QoL assessment provides a format to express whether an intervention has made an important difference to the patient’s life, but its measurement is a challenge in AD [20],[21],[28],[47]. Indeed, the abstract nature of QoL limits its use to individuals who have the cognitive capacity to understand the concept, a capacity that is gradually lost in neurodegenerative diseases such as AD (for example, the comprehension of the item ‘you as a whole’ or ‘you in general’) [21]. For this reason, self-rating of the QoL is often replaced by proxy rating. However, it has been shown that there could be differences on QoL ratings between patients, caregivers and theoretical models [48]. Caregivers consistently rate the patient’s QoL lower [14],[49], not only because of cognitive function [22],[50],[51] but also because of the caregiver’s burden or depression [22],[49],[50],[52],[53] and the patient’s depressive symptoms [54]. Acknowledging the problem of potential bias of proxy reports, we chose a proxy rating for QoL in the THERAD study, for several reasons. Firstly, it has been demonstrated with increasing severity of the disease that patient ratings must mostly be replaced by proxy ratings [55],[56], even if Logsdon and colleagues showed in 2002 that patients can rate their own QoL until the late stages [28]. This last study showed that the correlation between caregiver’s rating and patient’s rating was greatest for subjects in the middle tertile of cognitive function, and the recruitment of our centre for the THERAD study is mainly moderately affected patients. Then in our routine practice we found that it was difficult to estimate the patient’s QoL with self-rating. First, the patient’s ability to identify changes or to make choices among options in a scale is affected by lack of insight, due to anosognosia [30],[54]. Moreover, cognitive impairment, with varying deficits of memory, attention, judgement, insight and communication, influences the ability of individuals to comprehend questions or make comparisons in complex domains [47]. Then, at the moderately severe stage of the disease, patients must receive important help from the rater to complete the scale, which can introduce a real bias – the experimenter’s bias. For all these reasons we chose to use the caregiver’s rating of the AD patient’s QoL. We also decided to add self-rating methods as a secondary outcome in the THERAD study, because it has been demonstrated that self-rating and caregiver’s ratings are complementary and should be treated separately [20].

During the follow-up visits, we check the influence of the intervention on the carer’s perception of QoL by collecting, at each visit, data concerning his/her burden and depression. Indeed, burden and depression have been pointed out to influence the caregiver’s rating of the QoL [22],[49],[50],[52],[53]. We also collect data regarding patient’s cognition, which can also influence the caregiver’s rating of the QoL. The intervention in itself is designed to improve caregivers’ knowledge and to modify their attitude or behaviour, but not to specifically decrease their burden or improve their mood. Furthermore, psychosocial interventions have shown mild to modest efficacy in mitigating caregiver burden in high-quality meta-analyses. Many studies showed improvements in caregiver burden-associated symptoms (for example, mood, coping, self-efficacy) even when caregiver burden itself was minimally improved [57]. We thus hypothesised that the potential effect of our intervention would not be linked to a lesser caregiver’s burden that might modify his/her assessment of the patient’s QoL, but to an improvement in the patient’s QoL itself. There are similar data regarding patient’s depression [58] and cognitive function outcome, on which psychoeducational interventions have not demonstrated any positive impact [1]. Lastly, psychoeducational intervention providing caregiver education can decrease behavioural and psychological symptoms of dementia [13],[59] but these symptoms have not been correlated with the caregiver’s rating of the QoL [60].

The THERAD study follows a feasibility study we conducted at Toulouse University Hospital between January 2010 and January 2011. This was a 12-month monocentric quasi-experimental before and after study. The inclusion criteria were the same as those of the THERAD study. The main outcome was the patient’s QoL as assessed by the caregiver. Our secondary outcomes were: patient’s functional autonomy assessed by the Activities of Daily Living and the caregiver’s burden assessed by the Zarit Burden Interview. Twenty-nine dyads of AD patient/caregiver were included. At 2 months there was a significant increase in the patient’s QoL (24.6 ± 5.1 at M0 vs. 27.2 ± 6.0 at M2, *P* = 0.038). This was an encouraging result and the first step in the development of the THERAD study. Of course, this study had limitations, mainly its lack of statistical power. Use of the caregiver’s rating of the AD patients QoL can also be considered a limitation, in a combined intervention programme, but this limitation appears to be minimal. Indeed the educational programme was not specifically designed to improve the caregivers’ burden or depression, factors that have been pointed out to influence caregiver’s perception of the patient’s QoL. However, acknowledging this problem of proxy rating, we chose to add self-rating methods as a secondary outcome in the THERAD study. Moreover, it has been demonstrated that self-rating and caregiver’s ratings are complementary and should be treated separately [20]. This improvement in patients’ QoL was in accordance with the evidence from the literature. As mentioned earlier, a systematic review has shown that multidimensional approaches including, but not involving only, therapeutic education, caregiver support and respite improve the patient’s QoL [23]. In addition, it has been shown that therapeutic educational programmes can improve patients’ QoL in many chronic diseases [61],[62].

However, our feasibility study showed no significant differences in patients’ functional autonomy or caregivers’ burden over time. This is also consistent with the results in the literature. Indeed, the randomised controlled AIDMA study (psycho-educational programme assistance to caregivers of Alzheimer’s patients) [3] has not demonstrated any positive result on the patient’s functional independence measured by the Disability Assessment for Dementia [63]. In our opinion, an improvement in functional autonomy did not constitute an appropriate primary outcome measure for this type of intervention, in the light of the disease’s evolution. This study has, by contrast, shown an increased ‘sense of competence’ of the caregiver measured by the Sense of Competence Questionnaire [64]. Given the evidence from the literature and our daily experience, we hypothesise in the THERAD study that therapeutic education of both primary caregivers and AD patients could improve the AD patient’s QoL. If the efficacy of this type of approach is proven, and persists in the year after inclusion, then it will be important to implement such programmes in the care plan of AD patients.

## Conclusion

The THERAD study is the first trial designed to assess the specific impact of a therapeutic educational programme on the AD patient’s QoL. By helping AD patients’ caregivers to develop coping strategies and to increase their knowledge of the disease, we believe that the patient’s QoL could improve. If so, a corollary benefit would appear, such as a reduction in hospitalisations – which are frequent in this population, particularly in emergency departments [65]. The final results of this study may indicate whether such an approach needs to be implemented in the care plan of AD patients.

## Abbreviations

AD: Alzheimer’s disease

M0: baseline visit

M2: 2-month visit

QoL: quality of life

QoL-AD: Quality of Life in Alzheimer’s disease

THERAD: Therapeutic Education in Alzheimer’s Disease

## Competing interests

BV is a board member for Astra, Eisai, Elan, Exhonit, GSK, Lilly, Medivation, MSD, Nestlé, Nutricia, Pfizer, Pierre-Fabre, Roche, Sanofi, Servier, TauRx Therapeutics and Wyeth; and received grants from Avid, BMS, Elan, Exhonit, GSK, Ipsen, Lilly, Medivation, Pfizer, Pierre-Fabre, Roche, Servier, TauRx Therapeutics and Wyeth.

## Authors’ contributions

HV was responsible for the concept of the study and preparation of manuscript. VG was responsible for the methodological design of the study and analysis of data. AP was responsible for acquisition of subjects and drafting the manuscript. CH, SE and EdP were responsible for acquisition of subjects, collection of data and revision of the manuscript. AZ was responsible for collection of data, trial monitoring and revision of the manuscript. BV was responsible for supervision of the research group and revision of the manuscript. FN was responsible for correcting the manuscript, supervision of the study and revision of the manuscript. All authors read and approved the final version of the manuscript.
